# Supplementary material for: Enrichment of Wheat Bread with Platycodon grandiflorus Root (PGR) Flour: Rheological Properties and Microstructure of Dough and Physicochemical Characterization of Bread
Source: Foods. 2023 Jan 29;12(3):580. doi: 10.3390/foods12030580 (PMC9914062; doi:10.3390/foods12030580)
Supplement: Supplementary file 1 [file foods-12-00580-s001.zip › foods-2159850-supplementary.pdf]

SUPPLEMENTARY MATERIALS

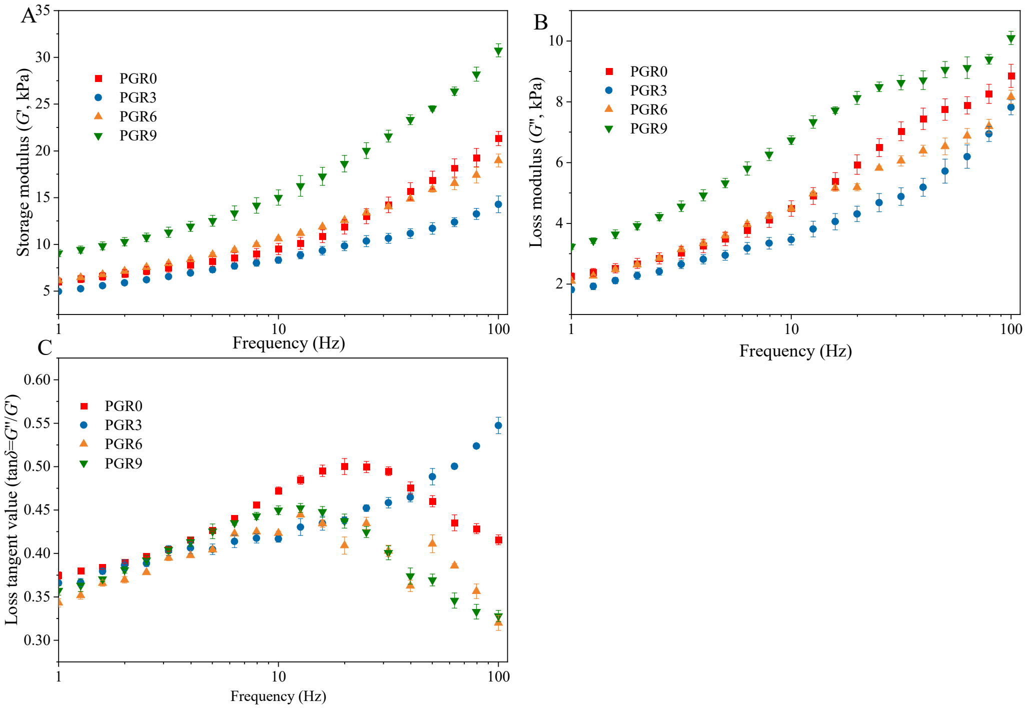

Figure S1. Rheological behavior curves of dough with different substitution levels of PGR flour. A:Storage modulus (*G*', kPa), B: Loss modulus (*G*'', kPa), C: Loss tangent values (tan *δ*)
